# The prevalence of musculoskeletal pain and therapy needs in adults with Osteogenesis Imperfecta (OI) a cross-sectional analysis

**DOI:** 10.1186/s12891-022-05433-3

**Published:** 2022-05-21

**Authors:** Sophie Barlow, Lucy Dove, Anju Jaggi, Richard Keen, Judith Bubbear

**Affiliations:** 1 Royal National Orthopaedic Hospital, Brockley Hill Stanmore HA7 4LP, Stanmore, England; 2 Oxford University Hospital, Oxford, England

**Keywords:** Osteogenesis imperfecta, Therapy, Physiotherapy, Mental health, Occupational therapy, Rehabilitation, Musculosketetal, Pain, Multidisciplinary

## Abstract

**Background:**

Osteogenesis Imperfecta affects approximately 1 in every 10,000 people. Musculoskeletal disorders and pain are common in adults with Osteogenesis Imperfecta, but specific knowledge of the problems people have is lacking. Access to therapy services for adults with Osteogenesis Imperfecta is variable. We designed this analysis to better understand the musculoskeletal disorders and consequent therapy needs for adults with Osteogenesis Imperfecta.

**Methods:**

This study was a cross-sectional analysis of outpatients with Osteogenesis Imperfecta. Adults attending a newly established multidisciplinary clinic at a tertiary centre in 2019 were included. A highly specialist physiotherapist worked within the clinic to offer therapy input if required and to refer patients to appropriate therapy as needed. People over the age of 18 were included if they had a diagnosis of Osteogenesis Imperfecta. Data were collected over a five month period using routinely collected clinical information and patient reported outcomes.

**Results:**

Over five months 50 patients attended the clinic. Musculoskeletal pain was a significant feature reported by 84% of patients. Over 50% of patients reported persistent pain for longer than one year duration and the most common site of pain was in the spine (46%). No difference in pain between types of OI and age. Forty five per cent (*n* = 19) of patients reported moderate to severe problems with mobility on the EQ-5D with over half reporting problems with self-care and ability to carry out usual activities. Over 50% of patients in clinic also reported anxiety (EQ-5D). During the consultation 70% of patients received therapy input which was either advice in clinic or an onward referral to the appropriate service. The referral rate to specialist out-patient rehabilitation services at a tertiary centre was 30%.

**Conclusions:**

This analysis highlights the high prevalence of MSK pain in adults with OI and the effect on physical function and emotional wellbeing. This study demonstrates the diverse needs of the adult Osteogenesis Imperfecta population and the need for suitable multidisciplinary therapy services.

## Background

Osteogenesis imperfecta (OI) comprises a heterogeneous group of genetic disorders characterized by increased bone fragility, low bone mass, and susceptibility to bone fractures with variable severity. OI affects approximately 1 in every 10,000 people [1] and in the UK, there are approximately 5,000 people living with OI. In 95% of cases the condition is caused by mutations in the COL1A1 and COL1A2 genes, and transmission is most commonly autosomal dominant. There are also autosomal recessive forms of the condition which generally have a more severe clinical presentation [2].

The clinical presentation of OI varies widely and is classified accordingly; mild (type I), neonatally fatal (type II), moderate deformity (type IV) and severe progressive skeletal deformity (type III) [2]. OI can affect multiple systems including cardiovascular, pulmonary, dental, auditory and musculoskeletal [3]. Bone fragility and recurrent fractures are the main clinical manifestation of the condition. Musculoskeletal (MSK) problems in OI could be due to joint hypermobility, scoliosis, deformity secondary to fractures, tendinopathy or muscles that fatigue easily. A recent study showed that adults with OI have a 64% rate of MSK concerns compared to 1–3% in a population without OI [3].

Management of OI should be multidisciplinary involving experienced medical, orthopaedic, physiotherapy and rehabilitation specialists. There is currently no cure for OI, although bisphosphonate therapy is used in children and can be used in adults [1]. The multidisciplinary approach to OI treatment is well established in the paediatric population however the transition to adulthood less so, despite concerns of other health conditions [3]. We therefore designed this analysis to understand what the MSK disorders and consequent therapy needs are of adults with Osteogenesis Imperfecta.

## Methods

In October 2019 a multidisciplinary team (MDT) clinic was established at a tertiary centre, for adults with OI. The team included two consultant metabolic bone specialists, a clinical nurse specialist, a geneticist, a research co-ordinator and a specialist physiotherapist with expertise in hypermobility and persistent pain. The project was reviewed by the local research and innovation centre at The Royal National Orthopaedic Hospital SE19.0035 and considered a service evaluation therefore no formal ethical approval was required. Routine clinic data was captured as part of the clinic appointment therefore verbal consent was obtained and recorded as part of patient notes. This was considered appropriate under the guidance of the research innovation centre.

The type of OI presentation was categorised by the consultants in clinic according to the Sillence classification [2] and whether they had a mild, moderate or severe phenotype.

STROBE standards for use in observational studies were used.

### Eligibility

Routine data was collected in the MDT clinic for patients who were over the age of 18 years and had a clinical diagnosis of OI.

### Data collection

Data were collected over a five month period from October 2019 to February 2020 by the physiotherapist in clinic. Demographic data including age, gender and work status were collected. Patient reported outcome measures EQ-5D-5L (EuroQol) [4] and brief pain inventory scale (short form) (BPS) [5] were collected by questionnaire. The patients were asked to complete the BPS based on their MSK pain. The BPS measures the severity of pain on an 11 point scale ranging from 0 (corresponding to ‘no pain’) to 10 (‘pain as bad as you can imagine’); we adopted the approach in a study by [6] Kawai et al. 2017 and took the average score as a measure of severity and categorized the scores as follows; mild pain (1–3), moderate pain (4–6) and severe pain (7–10). Pain interference with daily activities was defined as a mean score of 5 or above. In addition, we recorded the number of reported fractures in the last two years, the duration and site of MSK pain as well as the therapy intervention or onward referral.

### Data analysis

Descriptive data are given as means, ranges, frequencies and percentages. Excel version 14.7.3. was used to make calculations. Further statistical analysis was performed using Stata version 15.1. The comparison between the types of OI was performed using an un-paired t-test and the EQ-5D-5L outcomes were compared between groups using the Mann–Whitney test. An additional analysis examined the association between age and BPI pain score using a Pearson correlation.

## Results

During the five month data collection period fifty adults with OI attended the MDT clinic. The demographic data is presented in Table 1. The demographic data shows that more women than men accessed the clinic during this time whilst the prevalence in OI is equally split between genders. There were only a small number of type III patients, an insufficient number to include in a formal analyses between types. Thus, statistical comparisons were made between type I and type IV patients only.

**Table 1:** Characteristics of participants

	Total sample (*n* = 50)
**Age, mean (SD)**	38.42 (12.33)
**Age range (years)**	23–76
Female	31 (62%)
Male	19 (38%)
**OI Type**	
Type I	36 (72%)
Type III	4 (8%)
Type IV	10 (20%)
**Work status**	
Employed	27 (54%)
Not employed	17 (34%)
Studying	3 (6%)
Employed but on sick leave	3 (6%)

### Musculoskeletal pain and fractures

**Fig. 1:**
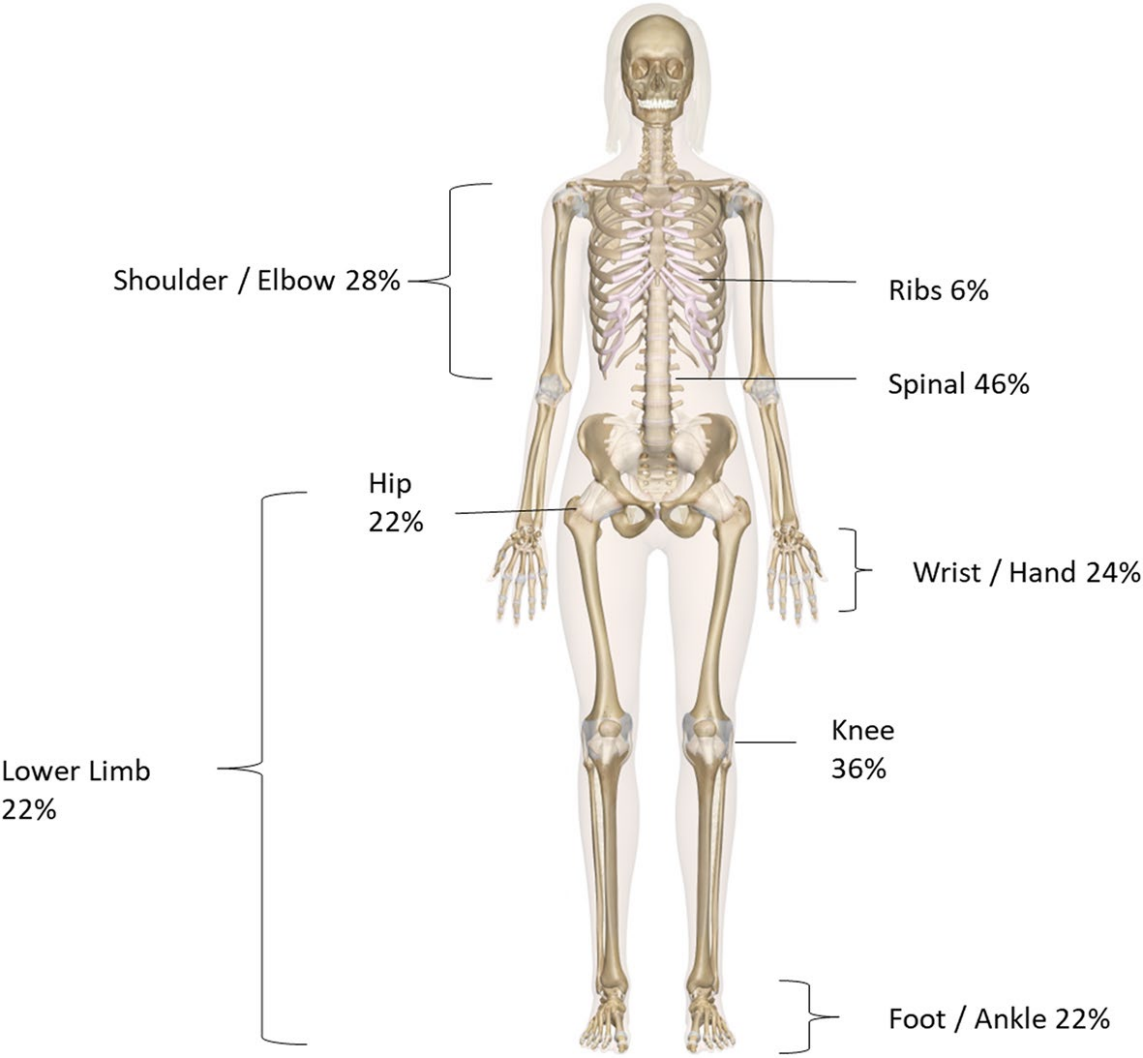
Reported sites of pain, more than one region could be selected so the sum of the numbers is greater than 100%

MSK pain was a significant feature of patients presenting in clinic with 86% (*n* = 43) of those attending reporting symptoms of pain. For those patients reporting pain, the duration of pain symptoms ranged from 0–12 months for (*n* = 14) of patients, between 1–2 years for (*n* = 12) of patients and (*n* = 17) of those in clinic reported having pain for over 2 years. The patients reported sites of pain in different areas of the body with (*n* = 14) reporting 1 site of pain, (*n* = 11) described 2 sites of pain and (*n* = 18) reporting 3 or more sites of pain. The distribution and frequency of the site of pain is shown in Fig. 1. Overall 27 of the patients reported 43 incidences of new fractures in the last 2 years.

The BPI severity scale was fully completed by 37 patients. This showed that 45% (*n* = 18) of patients have mild symptoms with a pain score of 3 or less, 35% (*n* = 13) have a pain score of 4–6 which was classified as moderate and 16% (*n* = 6) fell into the severe category with pain over 7/10. Overall 29% (*n* = 11) of patients had an average interference score of > 5 inferring that it impacted on their general activity, mood, walking ability, normal work, relations with other people, sleep and enjoyment of life. Statistical analysis demonstrated no difference in age (*p* = 0.82) or BPI pain scores (*p* = 0.20) when comparing the patients with OI type I and OI type IV. See Table 2.

**Table 2:** Comparison between OI types

Variable	Category	Type I (*n* = 26)	Type III (*n* = 4)	Type IV (*n* = 10)	*P*-value ^(*)^
Age	-	38.1 ± 11.2	35.0 ± 10.3	37.1 ± 12.8	0.82
BPI pain	-	3.8 ± 2.2	6.5 ± 1.0	4.8 ± 2.0	0.20
Equation 5d mobility	No problem	7 (27%)	0 (0%)	0 (0%)	** < 0.001**
	Slight problem	7 (27%)	0 (0%)	1 (10%)	
	Moderate problem	8 (31%)	0 (0%)	1 (10%)	
	Severe problem	4 (15%)	1 (25%)	5 (50%)	
	Unable	0 (0%)	3 (75%)	3 (30%)	
Equation 5d self-care	No problem	14 (54%)	0 (0%)	3 (30%)	0.08
	Slight problem	7 (27%)	0 (0%)	2 (20%)	
	Moderate problem	3 (12%)	1 (33%)	2 (20%)	
	Severe problem	2 (8%)	1 33%)	2 (20%)	
	Unable	1 (1%)	1 (33%)	1 (10%)	
Equation 5d usual activities	No problem	11 (42%)	0 (0%)	3 (30%)	0.32
	Slight problem	5 (19%)	0 (0%)	1 (10%)	
	Moderate problem	6 (23%)	0 (0%)	4 (40%)	
	Severe problem	3 (12%)	4 (100%)	0 (0%)	
	Unable	1 (4%)	0 (0%)	2 (20%)	
Equation 5d pain	No pain	3 (12%)	0 (0%)	0 (0%)	0.17
	Slight pain	9 (35%)	0 (0%)	2 (20%)	
	Moderate pain	8 (31%)	0 (0%)	5 (50%)	
	Severe pain	6 (23%)	2 (67%)	2 (20%)	
	Extreme pain	0 (0%)	1 (33%)	1 (10%)	
Equation 5d anxiety	Not anxious	10 (38%)	1 (25%)	6 (60%)	0.64
	Slightly anxious	8 (31%)	1 (25%)	1 (10%)	
	Moderately anxious	5 (19%)	1 (25%)	0 (0%)	
	Severely anxious	3 (12%)	0 (0%)	3 (30%)	
	Extremely anxious	0 (0%)	1 (25%)	0 (0%)	

*statistically significant

### Quality of life

**Fig. 2:**
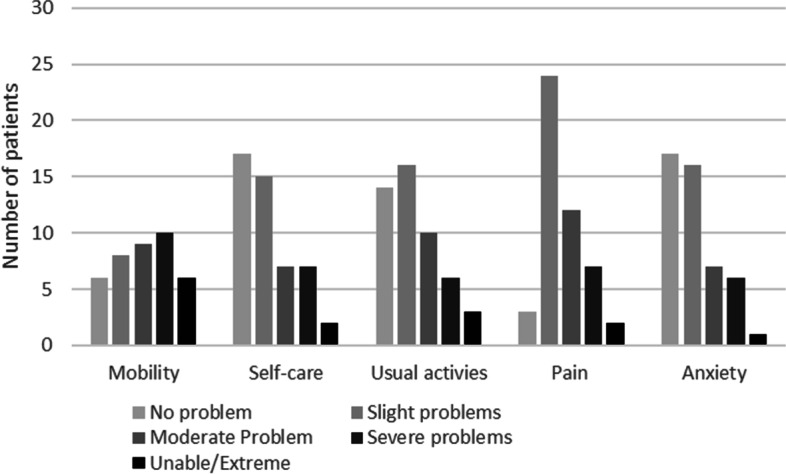
EQ5D data (*n* = 42)

The EuroQol EQ-5D-5L was used to measure overall quality of life. There were 42 full sets of data collected. One person declined to complete it, one was not collected and six were incomplete. Figure 2 (EQ5D data (*n* = 42)represents the data collected. In clinic 22% patients used a wheelchair as their primary mode of mobility, this included a self-propelling and powered wheelchairs, 18% of patients used a walking stick or crutches and 60% mobilised unaided. The mobility measure demonstrates that 45% (*n* = 19) of adults presenting in clinic have moderate-severe problems with their mobility and 14% (*n* = 6) reported they were unable to mobilise or had severe problems with mobility and 16% (*n* = 6) of patients reported no problems with their mobility.

Of the patients who completed the EuroQoL EQ-5D-5L reported pain 37/42 (88%). Slight pain problems were reported by 26% (*n* = 11) of patients and 33% (*n* = 14) reported moderate pain problems. Severe or extreme pain was reported by 28% (*n* = 12) of patients.

Of the completed measure just over half of the patients reported problems with managing self-care (52%, *n* = 22), 16% (*n* = 7) of patients reporting extreme to severe problems. However, 40% (*n* = 17) had no difficulties with self-care.

Regarding usual activities the data shows that 23% (*n* = 10) had moderate problems carrying out their usual activities whilst 23% (*n* = 10) had severe or extreme/unable to carry out usual activities and 33% (*n* = 14) did not report any difficulties with usual activities. Overall 54% (*n* = 23) reported anxiety. Of those patients, 24% (*n* = 10) reported slight problems, 14% (*n* = 6) reported moderate problems and one patient reported severe problems.

### Statistical findings according to type of OI

The EQ-5D-5L analysis suggest a statistically significant difference in mobility score between type I and IV patients (*P* = 0.01). The scores were higher for type 4 patients. 80% of type IV patients scored 4 or 5 on this scale, compared to only 15% of type I patients. See Table 2.

There was also some evidence that type IV patients had higher EQ-5D-5L self-care scores, although the difference for this measure did not reach statistical significance (*P* = 0.08). None of the other EQ-5D-5L scores varied significantly between the two groups. See Table 2.

### Therapy needs

**Fig. 3:**
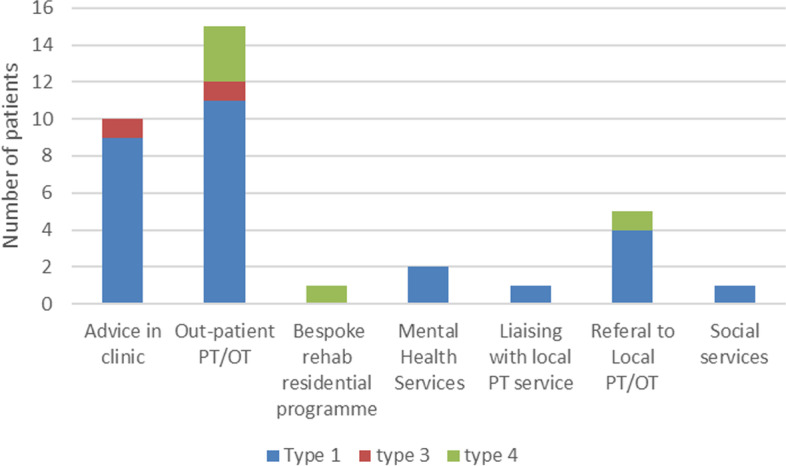
Therapy referrals and intervention in clinic

During the consultations, 70% of patients received therapy input which was either advice in clinic or an onward referral, Fig. 3. Half of the patients seen in clinic were referred to either physiotherapy, occupational therapy, social services or mental health services. Therapeutic advice was provided to 20% (*n* = 10) of patients in clinic, all of which were classified with Type I OI. A further 30% (*n* = 15) of patients were referred to out-patient rehabilitation services at a tertiary centre. A single patient with Type IV OI was referred to a bespoke rehabilitation in-patient stay at the same centre and a further 12% (*n* = 6) of patients were referred to their local community therapy service. One patient required a social services referral and 4% (*n* = 2) consented to onward referral for mental health services. The physiotherapist in clinic liaised with a local therapy team on behalf of one patient.

## Discussion

The data collected from the multidisciplinary clinic demonstrates the high prevalence of MSK pain in adults with OI and the consequent effect on daily function. MSK pain at numerous joints in the body is a key feature reported by this adult population and the results demonstrate that there was no correlation between the type of OI, age and the pain reported. Over half of patients in clinic reported persistent pain according to the International Association for the Study of Pain (ISAP) definition [7] A health survey in 2017 by Versus Arthritis found 34% of the population in England were suffering from chronic pain and the most common cause for pain was MSK conditions [8]. This suggests that adults with OI have a higher rate of chronic pain than the general population. Despite the reporting of new fractures in clinic, the incidence of persistent pain cannot be fully explained by current fractures and this has been reported previously by Lafage-Proust & Courtois [9].

It is widely understood that persistent pain adversely affects daily activities, physical and psychological health [6]. In a study by Tosi et al. [3] 95% of respondents with OI reported that MSK disorders affected their quality of life. A recent study by Nijhuis et al. 2021 [10] also finds that individuals with OI report pain as an important issue which impacts daily life, mobility, participation, work life and social relationships. The results from this study are in agreement with previous work, showing that that mobility, self-care and usual activity are impacted for over half of adults with OI who were seen in clinic. The significant difference in type IV mobility scores versus type I scores are not surprising as type IV OI is a physically more severe phenotype and clinically it is expected to affect their mobility. Despite a high percentage of patients experiencing pain and mobility difficulties, 60% of patients were still able to work or study (Table 1).

The most prevalent site of pain was the spine which may be explained by a high prevalence of spinal deformity such as scoliosis or vertebral fractures in the OI population [11]. Weight bearing joints in the lower extremity were also common as sites of pain and a study suggests joint instability and osteoarthritis can be a factor in these symptoms [12].

An interesting finding was just over half of patients reported anxiety symptoms on the EQ-5D-5L demonstrating that adults with OI may also suffer with mental health conditions. A systematic review by Tsimicalis et al. 2016 [13] supports this finding. The review reported that depression scores varied widely between individuals with OI and some studies showed reduced mental health and emotional functioning compared to a normative population. There is a consensus that emotional wellbeing should be part of a comprehensive assessment for individuals with OI [10] which further supports the importance of a biopsychosocial approach when assessing and treating adults with OI in a clinic environment.

The physiotherapist in clinic was able to provide advice within the clinic setting for 20% of patients. Within the clinic the physiotherapist was able to make an onward referral to the most relevant service based on the assessment, including psychology, occupational therapy, social services, physiotherapy or bespoke rehabilitation at a tertiary centre. Managing MSK disorders and long-term conditions holistically, has been advocated to reduce the impact of an individual’s disability and maximise their quality of life [14]. This cross sectional study demonstrates the need for multidisciplinary management in the adult population with OI which has long been established for children with OI.

## Limitations

This was a single centre study and data collection was curtailed as the clinic was suspended in March 2020 due to the Covid-19 pandemic. Due to this there was not enough data on type III OI patients to support statistical comparisons. Furthermore, incomplete data for outcome measures may have limited the results, this could be overcome if they were digital for any future studies in this area. We did not record the reasons or diagnosis of MSK pain as this was beyond the remit of this analysis but would be an interesting further study.

## Conclusions

This study highlights the high prevalence of MSK pain in adults with OI and the effect it has on their physical function and emotional wellbeing. The diverse needs of the adult OI population are demonstrated in terms of the wide variety of onward referrals required. Further work is required to understand the experiences of people with OI in accessing therapy services and their perceived therapy needs to support service design.

## Data Availability

The datasets generated and analysed during the current study are not publicly available due recognisable personal data but are available from the corresponding author on reasonable request.

## Abbreviations

OI: Osteogenesis Imperfecta
MSK: Musculoskeletal
MDT: Multidisciplinary team
ISAP: International Association for the Study of Pain
PT: Physiotherapy
OT: Occupational Therapy
